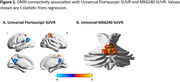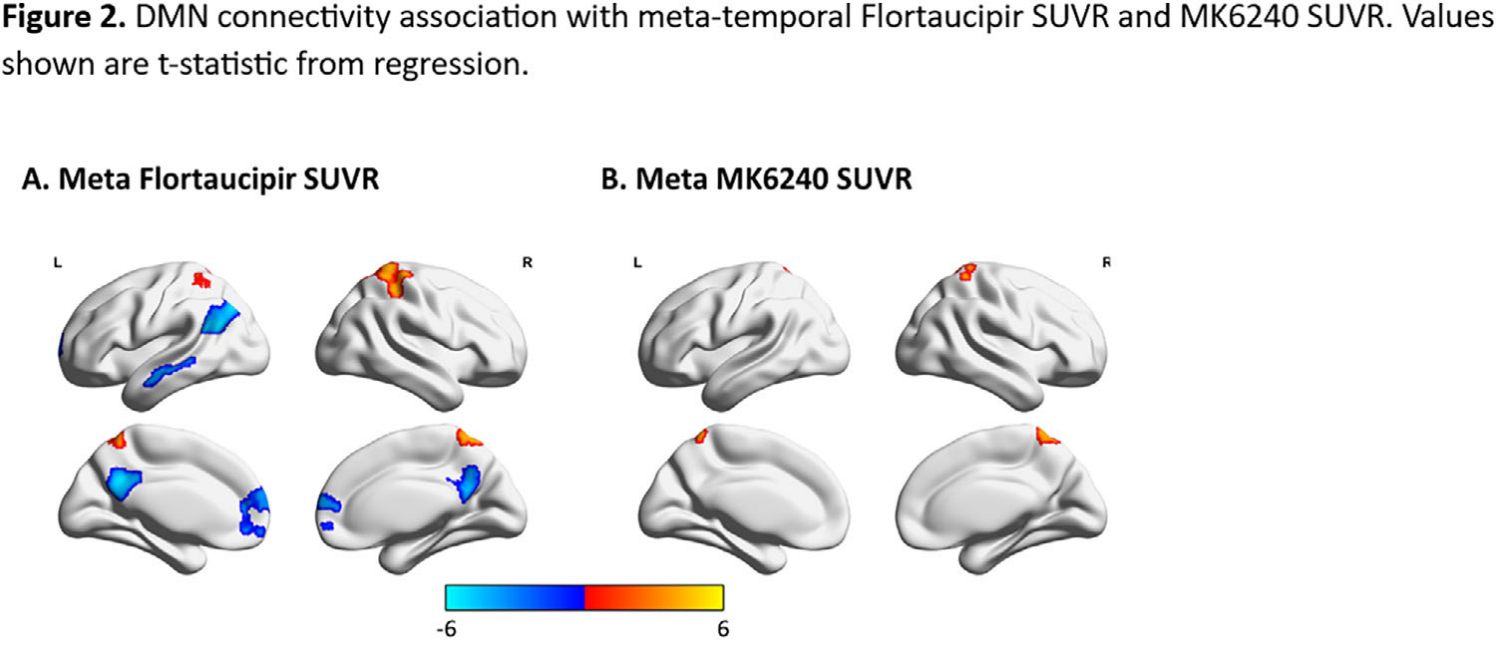# Association between Default Mode Network Connectivity Compared Between Two Tau Tracers (Flortaucipir vs. MK6240) – HEAD Study

**DOI:** 10.1002/alz.094117

**Published:** 2025-01-09

**Authors:** Helmet T. Karim, Guilherme Povala, Guilherme Bauer‐Negrini, Firoza Z Lussier, Belen Pascual, Brian A. Gordon, Val J. Lowe, Hwamee Oh, David N. soleimani‐meigooni, Pedro Rosa‐Neto, William J. Jagust, William E Klunk, Suzanne L. Baker, Tharick Ali Pascoal

**Affiliations:** ^1^ University of Pittsburgh, Pittsburgh, PA USA; ^2^ Houston Methodist Research Institute, Houston, TX USA; ^3^ Washington University in St. Louis School of Medicine, St. Louis, MO USA; ^4^ Department of Radiology, Mayo Clinic, Rochester, MN USA; ^5^ Brown University, Providence, RI USA; ^6^ Memory and Aging Center, Weill Institute for Neurosciences, University of California, San Francisco, San Francisco, CA USA; ^7^ Translational Neuroimaging Laboratory, Montreal, QC Canada; ^8^ University of California, San Francisco, San Francisco, CA USA; ^9^ Lawrence Berkeley National Laboratory, Berkeley, CA USA

## Abstract

**Background:**

Default mode network (DMN) resting state connectivity has been correlated with heightened amyloid and tau – hallmarks of Alzheimer’s Disease (AD). Tau is postulated to impact a meta‐temporal area including DMN‐associated regions like amygdala, entorhinal cortex, fusiform gyrus, parahippocampus, inferior temporal, and middle temporal gyrus. We recruited individuals with varying cognitive status to undergo resting state connectivity and imaging with two tau tracers (Flortaucipir and MK6240). We investigated the association between DMN connectivity and both global and regional (meta‐temporal) tau.

**Methods:**

We studied 149 adults who were young cognitively healthy (n = 10, 21‐25 years, 5F), older cognitively healthy (n = 85, 50‐83 years, 51F), had MCI (n = 43, 53‐84 years, 23F), or had AD (n = 11, 62‐84, 6F). All individuals underwent resting state imaging and PET imaging with both Flortaucipir and MK6240 tau tracers. Resting data was preprocessed and DMN connectivity was estimated using bilateral posterior cingulate gyrus as region of interest. Tau tracer uptake was preprocessed (Pascoal. et al), and voxel‐wise standardized uptake value ratio (SUVR) was computed using inferior cerebellar gray matter as reference. We computed universal and meta‐temporal uptake for both tracers. We conducted four voxel‐wise associations between DMN connectivity and uptake in each tracer. We corrected for multiple comparisons using SnPM13 to estimate non‐parametric p‐values (10,000 permutations), we used a cluster forming threshold of p = 0.001 and corrected for multiple comparisons by controlling the family‐wise error rate (FWE) at p = 0.05.

**Results:**

Greater universal Flortaucipir uptake was associated with lower within‐network DMN connectivity (bilateral posterior cingulate, precuneus, angular/supramarginal gyrus, and inferior parietal) but greater between‐network DMN connectivity (superior parietal, postcentral gyrus; Figure 1A). Greater universal MK6240 uptake was associated only with greater DMN‐cerebellum connectivity (Figure 1B). Greater meta‐temporal Flortaucipir was associated with lower within‐network DMN connectivity similar to universal uptake with addition of medial prefrontal cortex and middle temporal gyrus (Figure 2A). Greater meta‐temporal MK6240 was only associated with greater DMN‐superior parietal connectivity (Figure 2B).

**Conclusions:**

These preliminary findings show that uptake using different tau tracers may lead to varying conclusions on association with connectivity. This may have implications for understanding the role of tau in altering resting state connectivity findings.